# Unusual interplay of contrasting selective pressures on β-defensin genes implicated in male fertility of the Buffalo *(Bubalus bubalis*)

**DOI:** 10.1186/s12862-019-1535-8

**Published:** 2019-11-26

**Authors:** Vipul Batra, Avinash Maheshwarappa, Komal Dagar, Sandeep Kumar, Apoorva Soni, A. Kumaresan, Rakesh Kumar, T. K. Datta

**Affiliations:** 1 Animal Genomics Lab, National Dairy Research Institute, Karnal, 132001 India; 2 Theriogenology Lab, SRS of NDRI, Bengaluru, 560030 India

**Keywords:** Reproduction, Buffalo, Male fertility, Expression, Beta-defensin, Orthologs, Paralogs, Purifying and diversifying selection

## Abstract

**Background:**

The buffalo, despite its superior milk-producing ability, suffers from reproductive limitations that constrain its lifetime productivity. Male sub-fertility, manifested as low conception rates (CRs), is a major concern in buffaloes. The epididymal sperm surface-binding proteins which participate in the sperm surface remodelling (SSR) events affect the survival and performance of the spermatozoa in the female reproductive tract (FRT). A mutation in an epididymal secreted protein, beta-defensin 126 (DEFB-126/BD-126), a class-A beta-defensin (CA-BD), resulted in decreased CRs in human cohorts across the globe. To better understand the role of CA-BDs in buffalo reproduction, this study aimed to identify the BD genes for characterization of the selection pressure(s) acting on them, and to identify the most abundant CA-BD transcript in the buffalo male reproductive tract (MRT) for predicting its reproductive functional significance.

**Results:**

Despite the low protein sequence homology with their orthologs, the CA-BDs have maintained the molecular framework and the structural core vital to their biological functions. Their coding-sequences in ruminants revealed evidence of pervasive purifying and episodic diversifying selection pressures. The buffalo CA-BD genes were expressed in the major reproductive and non-reproductive tissues exhibiting spatial variations. The Buffalo BD-129 (BuBD-129) was the most abundant and the longest CA-BD in the distal-MRT segments and was predicted to be heavily O-glycosylated.

**Conclusions:**

The maintenance of the structural core, despite the sequence divergence, indicated the conservation of the molecular functions of the CA-BDs. The expression of the buffalo CA-BDs in both the distal-MRT segments and non-reproductive tissues indicate the retention the primordial microbicidal activity, which was also predicted by in silico sequence analyses. However, the observed spatial variations in their expression across the MRT hint at their region-specific roles. Their comparison across mammalian species revealed a pattern in which the various CA-BDs appeared to follow dissimilar evolutionary paths. This pattern appears to maintain only the highly efficacious CA-BD alleles and diversify their functional repertoire in the ruminants. Our preliminary results and analyses indicated that BuBD-129 could be the functional ortholog of the primate DEFB-126. Further studies are warranted to assess its molecular functions to elucidate its role in immunity, reproduction and fertility**.**

## Background

Beta-defensins (BDs), first discovered in *Bos taurus* [1], are highly conserved innate effector molecules present ubiquitously from the prokaryotes to the higher eukaryotes. This class of host defence peptides (HDPs) possesses antimicrobial activities against a variety of microbes ranging from the virus [2] and bacteria [3] to fungi [4]. The distinctive attributes of BDs are their relatively small size, the occurrence of six cysteine residues forming the three characteristic disulfide linkages, the presence of a defensin fold, and abundance of basic residues [5]. The disulfide bonds are formed between particular cysteines, C_1–5_, C_2–4_ and C_3–6_, and are required for CCR2-CCR6 (chemokine receptors) mediated chemotactic activities [6] usually displayed by these antimicrobial peptides (AMPs). The BD gene structure is usually comprised of two exons; exon 1 (E1), and exon 2 (E2), encoding for the signal and the mature peptide, respectively [1]. The similarities among the BD orthologs are apparent only at the structural level of their genes and/or their products, but not at the sequence level of their translated gene products. Many of the BDs are constitutively expressed in accordance with their pleiotropic functions [7] while others show ‘up-regulation’ during infection and in the presence of pathogens [8].

The BDs were hitherto known only as the innate effectors, the AMPs. Nonetheless, their emerging pleiotropic roles in immunomodulation [9], cancer [10], wound healing [11], cell migration [12], angiogenesis [13] and male reproduction [14, 15] are continually being reported. These AMPs are regulated majorly by the androgens and they link the innate and the adaptive immune systems by initiating crosstalk between these two arms of immunity [16]. They also have the ability to modulate the immune response towards pro-inflammatory Th1 or anti-inflammatory Th2 responses by modulating downstream signalling cascades of the chemokine receptors [17–19], and [20]. As previously mentioned, both the constitutive and the inducible expression of BDs has been reported [21, 22], and [23]. Some BDs are subject to positive regulation while others are negatively regulated [24, 25], and [16]. A number of BDs possess positive immune-modulatory properties, whereas others have negative modulatory roles [26, 27]. In addition to the orthologs, the paralogs also exist for BDs [28]. The single nucleotide polymorphism (SNP) genotypes of BDs have been shown to be associated with milk and meat yield [29, 30]. Further, their copy numbers are now well known to affect numerous traits, including biotic resistance, colour, yield etc. [31, 32].

**Fig. 1:**
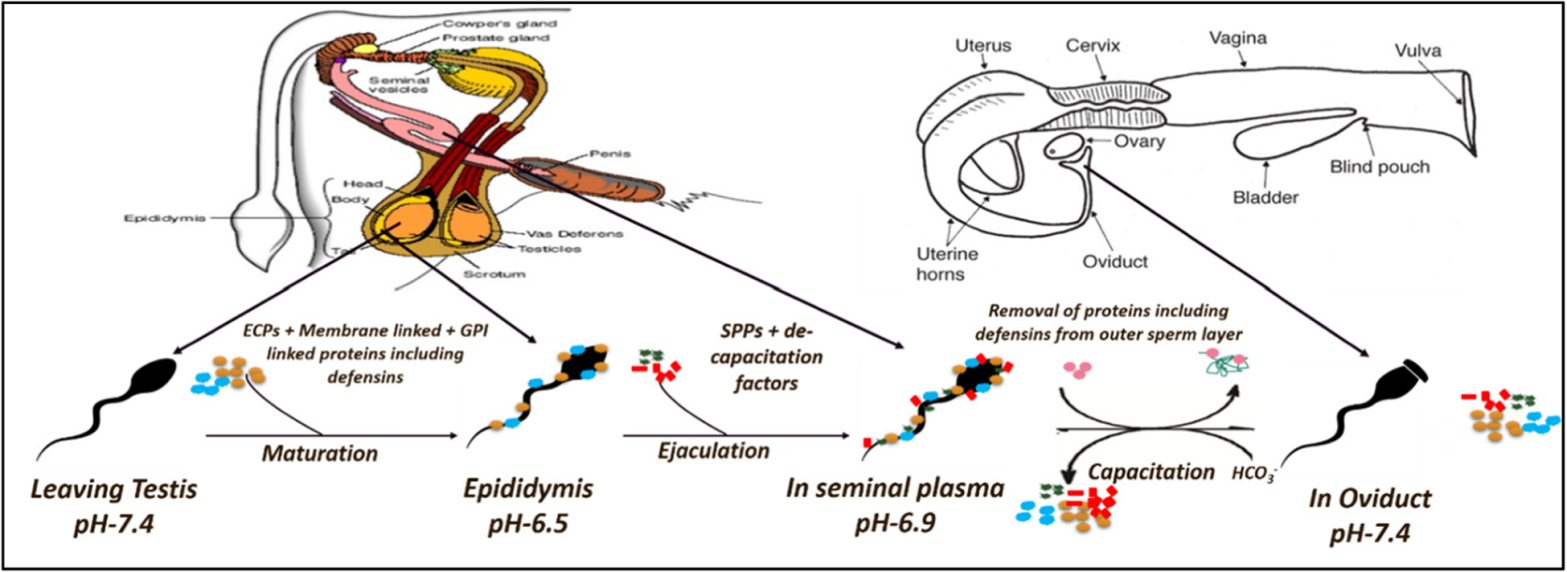
SSRs in the MRT

The pleiotropic roles of the BDs in male fertility and reproduction have gained considerable attention since Li et al. [33] reported for the first time about the role of a BD, Bin1b, in reproduction and reproductive tract (MRT) host defence. This BD was found to be present in the proximal-MRT segments where it assists the spermatozoa in their maturation and storage. In addition, it inhibits the invasion of microorganisms in the neighbouring gamete production site, the testes. Numerous reports have emerged about the preferential and spatiotemporal regulation of BD expression in the MRT [34, 35], and [36]. A number of secretagogues, like Bin1b and DEFB-126, are applied as a peripheral coat onto the sperm surface during its passage through the epididymis. Such a coat assists the spermatozoa in the FRT to ultimately fertilize the egg (Fig. 1).

Spermatozoa undergo extensive remodelling of their surface in the epididymis where several proteins are adsorbed, inserted and bound covalently or non-covalently on its surface. These include- i) the epididymal coat proteins (ECPs) which may be deep-seated in the plasma membrane via the hydrophobic interactions, ii) electrostatically bound proteins iii) Glycosyl-phosphatidylinositol (GPI) anchored proteins and proteins interacting with them iv) proteins in apical blebs known as epididymosomes that are embedded in the sperm plasma membrane and are usually trans-membrane. The seminal plasma proteins (SPPs) and the de-capacitation factors are finally added in the seminal fluid before ejaculation. In the FRT, the capacitation induced changes in the outer sperm membrane cause loss of many proteins e.g. DEFB-126, in a site-specific manner. (Adapted and modified from Rich TD, and Turman EJ.–Beef Cattle Handbook-2200) [37].

The secretagogues from the distal segments of the MRT include the products of a specific BD cluster (e.g. hBD-126 on human chromosome 20 and macaque DEFB-126 on chromosome 10), which are secreted and then adsorbed on the surface of the traversing spermatozoa [38]. These specific BDs were named class-A to demarcate them from other BD family members based on their chromosomal cluster, tail lengths, and their roles in male reproductive functions [30]. The functional roles played by these HDPs, especially the CA-BDs, in reproduction and fertility are diverse and equally complex [39, 40]. The CA-BD family members (e.g. DEFB-126) have been demonstrated to render the spermatozoa un-capacitated before they reach the site of fertilization [41]. Further, they assist the spermatozoa to surmount the arduous barriers in the FRT, such as cervical mucus [42], immune response by the PMNs and the macrophages in the uterus and other segments of the FRT [43]. These AMPs are also required for acquiring sperm motility in the epididymis prior to capacitation [14] and for the formation of the oviductal sperm reservoir [44]. The primate CA-BDs have also been demonstrated to assist the spermatozoa to capacitate and to finally attain their ultimate goal of fertilizing the ovum by playing a crucial role in the capacitation process and sperm-zona recognition during fertilization [45, 46]. Molecules with such disparate and critical roles need to be further explored and characterized for unveiling the complete diversity of their pleiotropic functions, especially in the reproductive processes.

The buffalo was considered as a model for this study due to its economic importance in the developing countries. Although it is a premier dairy animal with superior milk-producing ability, it suffers from certain reproductive limitations that constrain its lifetime productivity. Male sub-fertility which is often manifested as low CRs is a major concern in buffalo. Interestingly, a mutation in defensin 126 (DEFB-126) was found to be responsible for decreased CRs in human cohorts across the globe [38]. However, the role of BDs in buffalo reproduction and reproductive tract defence is unknown. Therefore, the objectives of the present work were to characterize the buffalo BD gene clusters and their products with special reference to CA-BDs, to understand the selection pressure(s) on these genes, to identify the most highly expressed CA-BD gene in the buffalo MRT, and to predict its reproductive functional significance.

## Results

### In silico sequence analyses of the buffalo CA-BDs

**Fig. 2:**
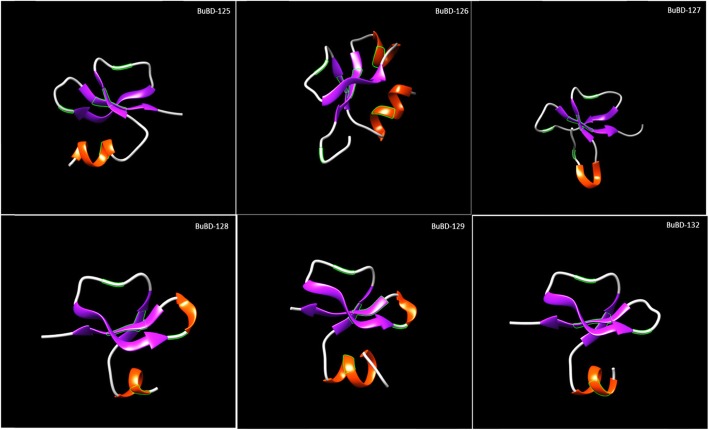
Putative 3D structures of BuBDs

Thirty BD genes, including 15 novel and the six CA-BDs, were mined from the buffalo reference sequence (RefSeq) assembly (UOA_WB_1) based on their size, sequence, gene structure and cluster similarities with that of cattle BD orthologs. Similarly, the BD genes were mined from the RefSeq assemblies of other ruminant, pseudo-ruminant and non-ruminant species (Additional files 1 and 2). The BDs in buffalo were found to be located in four clusters on the chromosome No. 1, 2. 3 and 14. The standardized gene nomenclature was used for naming the identified buffalo BDs as BuBDs on the basis of their orthologous relationship with that of the cattle and the considered mammalian species. The size of the class-A BuBD cluster was found to be approximately 120 kb on the chromosome 14 *vis-a-vis* 320 kb on the chromosome 13 of the cattle [30, 48]. Moreover, all the retrieved BDs exhibited strong evidence of synteny. The six class-A BuBDs which were considered for further analyses were found to be oriented in either 5′-3′ (plus) or 3′-5′ (minus) direction (e.g. BuBD-128). In addition, other classical AMPs like the Lingual Antimicrobial Peptide (LAP), Tracheal Antimicrobial Peptide (TAP), Bovine neutrophil BDs (BNBDs) and Enteric BD (EBD), were also retrieved from the buffalo RefSeq assembly. Further analysis of these classical AMPs was excluded as they are known to be well-characterized as AMPs [47],

The translated gene products of the identified BuBD cluster on chromosome 14 (i.e. CA-BDs) were subject to in silico sequence analyses, which revealed the conservation of the key BD molecular framework characterised by the six cysteine motif and the hallmark spacing between the cysteines similar to their identified orthologs in other mammalian species (Additional file 3). Interestingly, the multiple sequence alignment (MSA) of class-A BuBDs revealed the presence of unique gene-specific motifs (GSMs), which were either present in all the considered species or only ruminant species (Additional file 3). For example, the ERY motif in BuBD-125 and QVNT motif in BuBD-129 are GSMs present in all the species, whereas SSCIPL motif in BuBD-125 and MMQT motif in BuBD-129 are GSMs limited to the ruminants. The effect of substitution with other amino acids at each site also determined that the first residue of the BD peptide, the six cysteines and their immediate neighbouring residues were the most crucial residues to the function of class-A BuBDs. Thus, the strongest signals (score > 50) were produced for their predicted effect by the SNAP2 tool (Additional file 3). Further, all the class-A BuBDs were found to contain abundant positively-charged amino acids, lysine (K) and arginine (R), and polar residues like threonine (T) and serine (S) (Additional file 3). The loops were found to be the major secondary structural elements of the class-A BuBDs, as predicted by PROFsec. Most of the region, of the class-A BuBD proteins was predicted to be exposed and hence solvent accessible (Additional file 3). Nevertheless, the beta*-*strands and the alpha helices were also predicted, albeit in lesser proportions. In addition to the above secondary structural elements, the Meta disorder predictor identified the presence of long regions with no regular secondary structure (NORS) i.e. Seventy or more consecutive surface residues without either the beta strands or the helices in all the class-A BuBDs (Additional file 3). As expected, the putative 3D-structures of their translated gene products were found to contain loops in a major region. The 3D-structures of the modelled buffalo CA-BDs also contained the characteristic three β strands in the anti-parallel beta-sheet arrangement and a flanking partial helix at the N-terminus (Fig. 2). All the modelled class-A BuBDs exhibited a high similarity in their 3D-structures. The BuBDs appear to be secreted in response to either biotic or abiotic stimuli and exhibit binding and regulatory activities with relatively high reliability score as predicted by the Metastudent tool (Additional file 3). The ProfISIS and someNA tools predicted BuBD-125 to be involved in nucleotide binding between the amino-acid residues 32–37, whereas three BuBDs viz. BuBD-125, BuBD-127, BuBD-128 were predicted to be involved in protein-protein interactions between the amino acids 32–40, 32–47 and 28–35, respectively (Additional file 3). Furthermore, all the class-A BuBDs were predicted as AMPs by using a support vector machine (SVM) classifier computational strategy. The primate DEFB-126 and its murine ortholog are heavily glycosylated proteins possessing nine and 13 O-glycosylation sites respectively [30, 49]. O-glycosylation is a crucial post translational modification required for the pleiotropic functions performed by the CA-BDs e.g. cervical mucus penetration (CMP). However, amongst the class-A BuBDs, BuBD-129 was predicted to be heavily glycosylated, possess ten O-glycosylation and three N-glycosylation sites and BuBD-125 was predicted to contain seven O-glycosylation sites (Table 1). Intriguingly BuBD-126, the sequence ortholog of DEFB-126 had only two O-glycosylation sites and a trans-membrane helix as predicted by the NetOGlyc 4.0 and TMHMM server *ver.* 2.0, respectively.

**Table 1:**
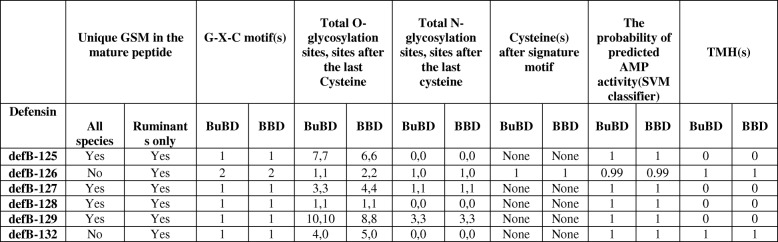
In silico prediction for the various sequence characteristics of the buffalo CA-BDs vis-à-vis bovine CA-BDs

The class-A BuBDs were modelled on the available structures of BDs, in the protein data bank (PDB), using the SWISS-MODEL server. BuBD-125,126 and 132 were modelled on human beta defensin 6, hBD6 (2lwl.1.a), BuBD-127 on hBD4 (5ki9.1.A), BuBD-128 on hBD1 (1kj5.1.a) and BuBD-129 on the bovine neutrophil defensin BNBD (1bnb.1.a) All the defensins possess the characteristic β-fold despite the low homology amongst orthologs**.**

### Evolutionary analyses

#### Phylogenetic tree

**Fig. 3:**
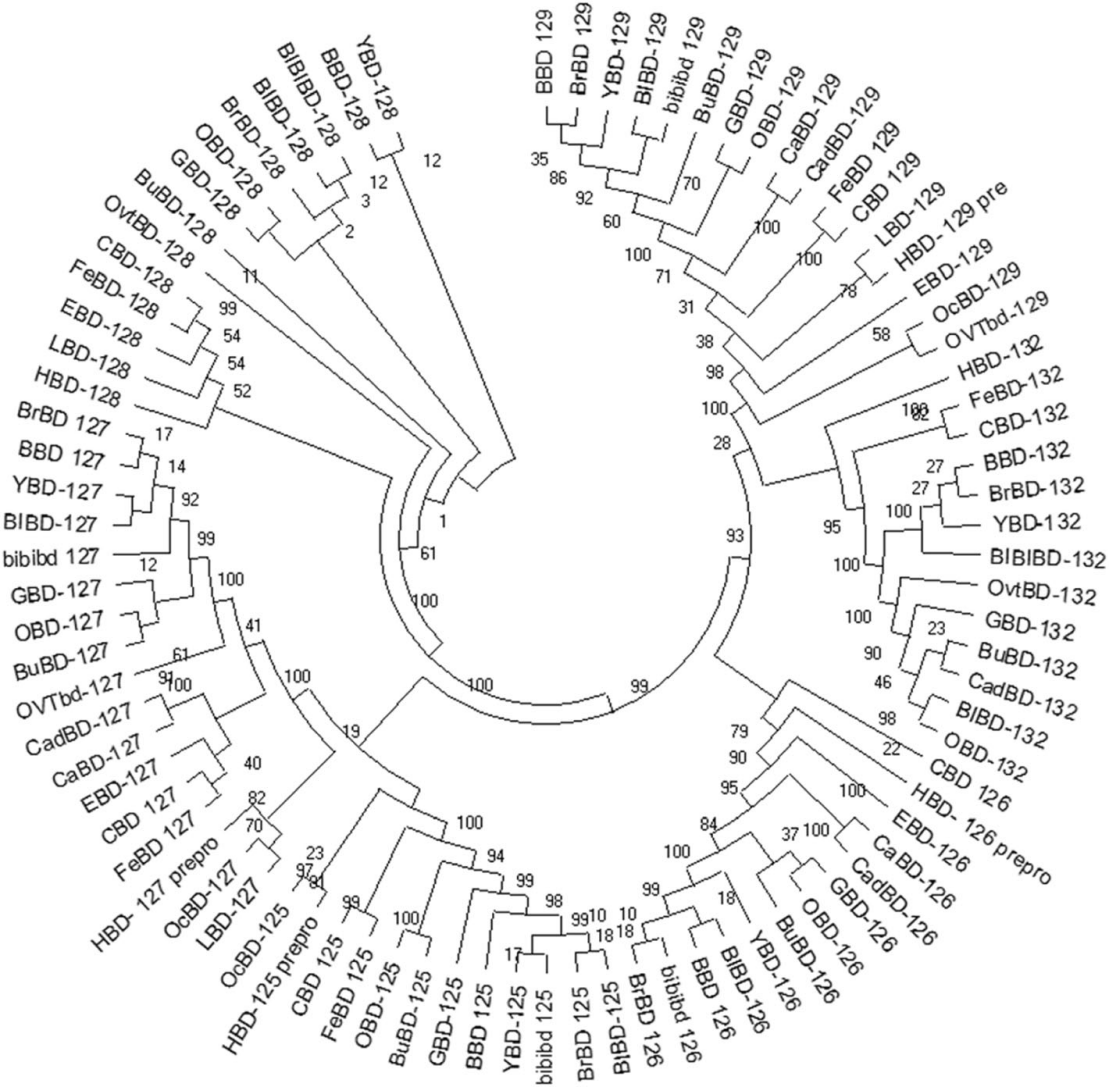
Evolutionary analysis by Maximum Likelihood method

The consensus tree indicated a strong evolutionary relationship between the buffalo CA-BD genes and their orthologs in other ruminant, pseudo-ruminant and non-ruminant species (Fig. 3). These defensin family members in buffalo have maintained a clear 1:1 orthologous relationship with BDs in their respective orthologous families, and they were found to be clustered accordingly. Interestingly, the BuBDs-125, 126, and 127 sub-clustered with ovine or caprine branches, whereas the BuBD-128, 129 and 132 segregated from *Bovidae* sub-sub cluster. Moreover, BuBD-128, 129 and 132 appeared to be more evolutionary related to the indicine than the taurine cattle.

The bootstrap consensus tree was inferred using the Maximum Likelihood method and the JTT matrix-based model from the translated coding sequences (tr-CDSs) of CA-BD genes. The bootstrap consensus tree inferred from 1000 replicates is shown.

### Selection pressure characterization

Various statistical models and tools in the HyPhy (Hypothesis testing using Phylogenies) package determined that episodic and pervasive contrasting selective pressures acting on the BD genes have expanded and maintained the BD functional repertoire in the buffalo and in the other ruminants. This temporal orchestration of the contrasting selective pressures has endowed the BDs with functional diversity and the ability to preserve the novel acquired functions.

### Analysis of selection pressure on the CA-BD orthologs by HyPhy

The GARD (Genetic Algorithm for Recombination Detection) analysis of the CA-BD orthologs from the 17 different non-ruminant, pseudo-ruminant and ruminant species, including the buffalo, revealed the presence of the two putative recombination breakpoints in the exon2 (E2) alignment of BD-128 and 129 only. However, both were found to be non-significant to produce a topological incongruence by the KH test (Kishino–Hasegawa test, at *P* = 0.05). No recombination breakpoints were found in other CA-BDs. The selection pressure was subsequently characterized at the branch and the site levels by selecting the buffalo or all true ruminants *‘*a priori*’* as the foreground branches for analyses (Table 2 and 3)*.* The aBSREL (adaptive branch-site random effects likelihood) tool didn’t reveal any evidence of the episodic selective pressure acting on the exon1 (E1), exon2 (E2) and the whole-CDSs either in the ruminants or the buffalo. However, RELAX revealed an ineffable intensification of positive selection only on the whole-CDS of BuBD-125 and E2 of BuBD-127 in the buffalo and other true ruminants despite the fact that no episodic selective pressure was found at any of the branches of the CA-BDs. The MEME (mixed effects model of evolution) tool detected the strong episodic site-level diversifying selection acting on the E2 and the whole-CDS of the ruminant and buffalo BD-125, 126, 128 and 129. The BD-125 and 129 had 13 and 6 codon sites under such selective pressures both in the ruminants and the buffalo (at *P* = 0.05). No episodic diversifying selection was, however revealed in the E1 of any of the ruminant or buffalo BDs. The BUSTED (branch-site unrestricted statistical test for episodic diversification) tool revealed the gene-wide evidence of episodic diversifying selection only in whole-CDS of ruminant BD-127 (*P* = 0.026) and the E1 of BuBD-126 (*P* = 0.006). Taken together, ample evidence of site-level episodic diversifying selection in the CA-BDs was revealed in the ruminant and buffalo CA-BD gene elements. Nonetheless, as determined by the fixed effect likelihood (FEL) tool, the E1 of ruminant and buffalo BD-126 had more sites under pervasive diversifying selection, while the E1 of ruminant BD-132 was under purifying selection. In ruminants, the E2 of BD-125 and 132 had four and three codon sites respectively, under pervasive purifying selection (at *P* = 0.05) in contrast to the buffalo where BuBD-126 was the only CA-BD under pervasive purifying selection. Additionally, all the ruminant CA-BD CDSs, except the ruminant BD-127, had at-least one codon site under pervasive purifying selection (at *P* = 0.05). However, two codon sites of the ruminant BD-125 and no sites of the BD-132 were under pervasive diversifying selection (at *P* = 0.05). Likewise, the E1 of BuBD-126 (3 codon sites) and 132 (1 codon site) and the E2 of BuBDs-125 (four codon sites) and BuBD-126, 127 & 129 (one codon site each) revealed the evidence of site-level pervasive diversifying selection (*P* = 0.05).

**Table 2:**
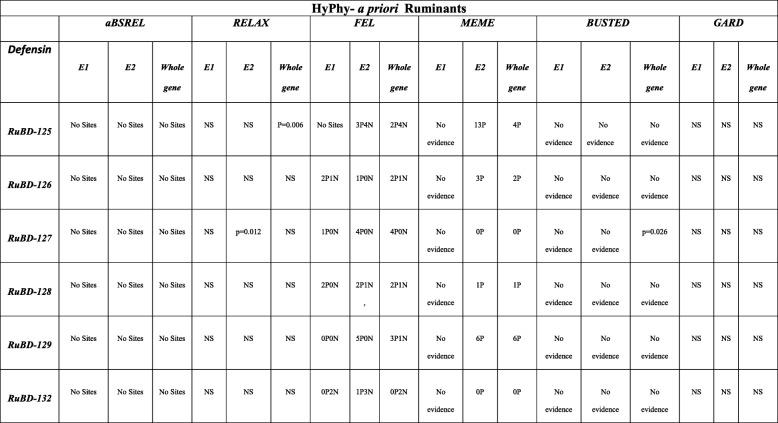
Compiled results of the various statistical tools, used to characterize the selection pressure in the CA-BD genes amongst the ruminants selected ‘a priori’ as the foreground branches, as implemented on the datamonkey server, a graphic user interface (GUI) for HyPhy (Hypothesis testing using Phylogenies). NS = non-significant, xPyN (for FEL) = x codon sites under positive and y under negative selective pressure respectively, RuBD = Ruminant beta-defensin

**Table 3:**
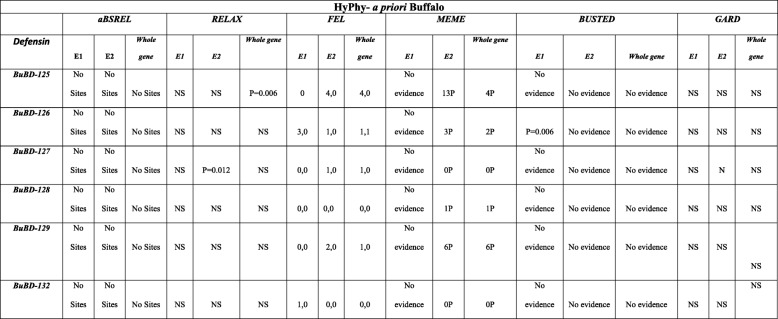
Compiled results of the various statistical tools, used to characterize the selection pressure in class-A beta-defensin genes in the buffalo selected ‘a priori’ as the foreground branch, as implemented on the datamonkey server, a GUI for HyPhy (Hypothesis testing using Phylogenies). NS = non-significant, xPyN (for FEL) = x codon sites under positive and y under negative selective pressure respectively, BuBD = Buffalo beta-defensin

### Analysis of selection pressure on the CA-BD orthologs by molecular evolutionary genetic analyses (MEGA-X)

The MEGA-X software detected the evidence of only purifying selection acting on CA-BDs, which appeared to be the major force in shaping the BD evolution in ruminants under dissimilar substitution patterns. The substitution patterns of the sequences were found to conform to homogeneity in the E1 of all the CA-BDs (Table 4) across the selected mammalian species. This suggested that these sequences have evolved with the same pattern of substitution, as adjudged from the extent of differences in base composition biases between sequences. However, the test of the homogeneity showed a significant (*P* < 0.05) difference in the substitution patterns of the E2 and whole-CDSs among all the CA-BDs, except for BD-128 and 132. The probability values from the one-tailed Fisher’s exact test of neutrality were non-significant for the sequence pairs of both the exons as well as the whole-CDSs of all the CA-BDs indicating the possibility of negative or positive selection on these gene elements. However, the codon-based test for analysis between the sequences revealed no evidence of positive selection (*P* > 0.05) on the E1, E2 and the whole-CDSs. Nevertheless, the codon-based tests considering all the sequence pairs together determined a significant excess of synonymous substitution per site (dS) over non-synonymous substitutions per site (dN), (dS > dN,*p* = 0.01) in most of the E2 and whole CDSs of all the considered CA-BDs, except BD-132. Tajima’s D was found to be negative for whole-CDSs of most of the CA-BDs, except for the BD-128. Thus, the substitution patterns acting on BDs appear to be non-neutral and such dissimilar substitution patterns are indicative of directional selection.

**Table 4:**
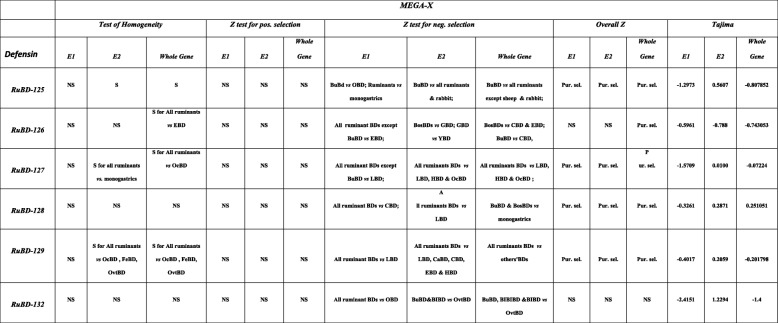
Compiled results of the various tests characterizing the selective pressures in the CA-BD genes in buffalo, as implemented in MEGA-X. NS = non-significant, S = significant, Pur. sel. = Purifying selection, RuBD = Ruminant beta-defensins

### Analysis of selection pressure on the CA-BD paralogs by HyPhy

HyPhy analyses indicated that a strong pervading purifying selection interwoven with the episodes of diversifying selection has acted on the CDSs of the buffalo paralogs. As observed in the orthologs**,** aBSREL analysis (Table 5) was not able to detect any evidence of episodic selective pressures on any of the branches of the exons and the whole-CDSs of the 30 BuBD paralogs. However, MEME detected episodic diversifying selective pressures across the E2 and the whole-CDS, but not in the E1 of these paralogs. The presence of gene-wide incidences of diversifying selection acting on the E2 (*P* = 0.023) and whole-CDSs (*P* = 0.000) of BuBD paralogs was revealed by the BUSTED tool. However, the results were non-significant if the class-A BDs were selected ‘a priori*’* as foreground branches to be tested.

**Table 5:**
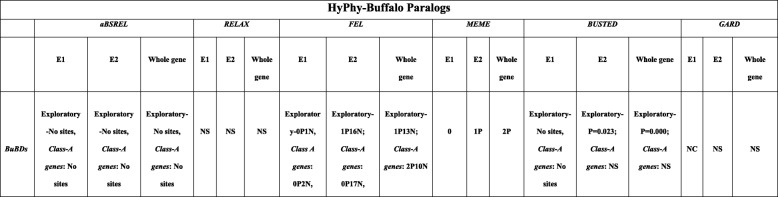
Compiled results of the various tools, used to characterize the selection pressures in BuBD paralogs of the buffalo in exploratory mode and ‘a priori’ with the CA-BD genes selected as the foreground, as implemented on the datamonkey server, a GUI for HyPhy. NS = non-significant, xPyN (for FEL) = x codon sites under positive and y under negative selective pressures respectively, BuBD = buffalo beta-defensin

The FEL results revealed that the E1 of all BuBD paralogs had one codon site, while the E1 of the CA-BDs had two codon sites under pervasive purifying selection (at *P* = 0.05). Similarly, the E2 of all BuBDs had 16 codon sites and the E2 of class-A BuBDs had 17 codon sites under pervasive purifying selection (at *P* = 0.05). Thirteen codon sites were determined under pervasive purifying selection in comparison to a single codon site under pervasive diversifying selection in the whole-CDSs of the BuBD paralogs (at *P* = 0.05). The class-A BuBDs also exhibited a similar pattern with 10 codon sites under the pervasive purifying and 2 codon sites under the pervasive diversifying selection (at *P* = 0.05).

### Expression dynamics of the CA-BD genes across the MRT and other tissues

The relative expression profiles of the selected panel of class-A BuBD genes were generated using qRT-PCR, in the five different segments of the MRT viz. Rete testis, the caput, corpus and, the cauda of epididymis and vas deferens (Additional file 3) and six non-reproductive tissues viz. Brain, heart, spleen, bladder, liver and lung from buffalo bulls, and two reproductive tissues from the FRT, the uterus and ovary. The GAPDH (glyceraldehyde-3-phosphate dehydrogenase) and eEF-2 (eukaryotic elongation factor) were employed as the reference genes, and the rete testis was considered as the calibrator tissue since the expression of the BDs, especially the CA-BDs is known to be absent in the rete-testis. The expression of BuBD-126 and a novel candidate BuBD-129 was found to be the highest in the distal segments of the MRT. A large variability in the expression levels in the MRT was observed among the biological replicates. In contrast to the cattle, the buffalo non-reproductive tissues actively expressed the CA-BDs although without much inter-animal variation**.**

### Expression of the class a BuBDs in the MRT

A high spatial and inter-animal variability was observed in the gene expression pattern of class-A BuBDs across the MRT. The highest expression of the class A BuBDs was found in the epididymis specifically the corpus epididymis, which also exhibited highest inter-animal variability in the expression levels. Primarily, a site-specific expression pattern of the class-A BuBDs was observed across all the segments of the MRT starting from the caput epididymis. The mean expression of BuBD-125 in the corpus epididymis was higher compared with the expression observed in either the caput (*P* > 0.05) or in the distal segments of MRT, the cauda (*P* < 0.01) and the vas deferens (*P* < 0.001). Interestingly, the ortholog of DEFB-126 in buffalo i.e. BuBD-126 was found to be preferentially expressed in the corpus and cauda epididymis and decreased later in the VD (*P* > 0.05). The expression level of BuBD-126 was significantly higher in the caudal region of the epididymis as compared to the caput epididymis (*P* < 0.05). On the contrary, BuBD-127 was preferentially expressed in the caput epididymis and diminishes thereafter. Its level of expression in the distal segments of MRT i.e. cauda and vas deferens was not significantly different from each other. For BuBD-128 the proximal regions i.e. the caput (*P* < 0.01) and the corpus epididymis were the preferred sites of expression, after which its expression decreased significantly in the cauda (*P* < 0.05) and VD (P < 0.01) when compared to corpus epididymis. The expression of BuBD-129 followed the expression dynamics of BuBD-126 with peak expression in the corpus epididymis (*P* < 0.001) and maintaining this elevated expression in the distal MRT segments. The coefficient of variation values indicated that the corpus epididymis exhibited the highest inter-animal variability in the expression levels of the BuBD-129 across the MRT. The BuBD-126 and BuBD-129 were the two class-A BuBDs with the highest expression levels in distal regions viz. the cauda epididymis and the vas deferens. BuBD-132 initially followed a similar trend in expression, however, its expression levels decreased in the distal MRT segments. As evident from the heatmap (Additional file 3) the relatively proximal parts of the MRT viz. caput and corpus epididymides lied in one cluster and the distal parts of the MRT cauda and vas deferens in a different cluster. It indicated that the mean expression profiles for proximal regions of the MRT were similar to each other like that of distal regions. However, the mean expression profiles of the proximal and distal region were very different from each other.

### Expression of the class-a BuBDs in other tissues

**Fig. 4:**
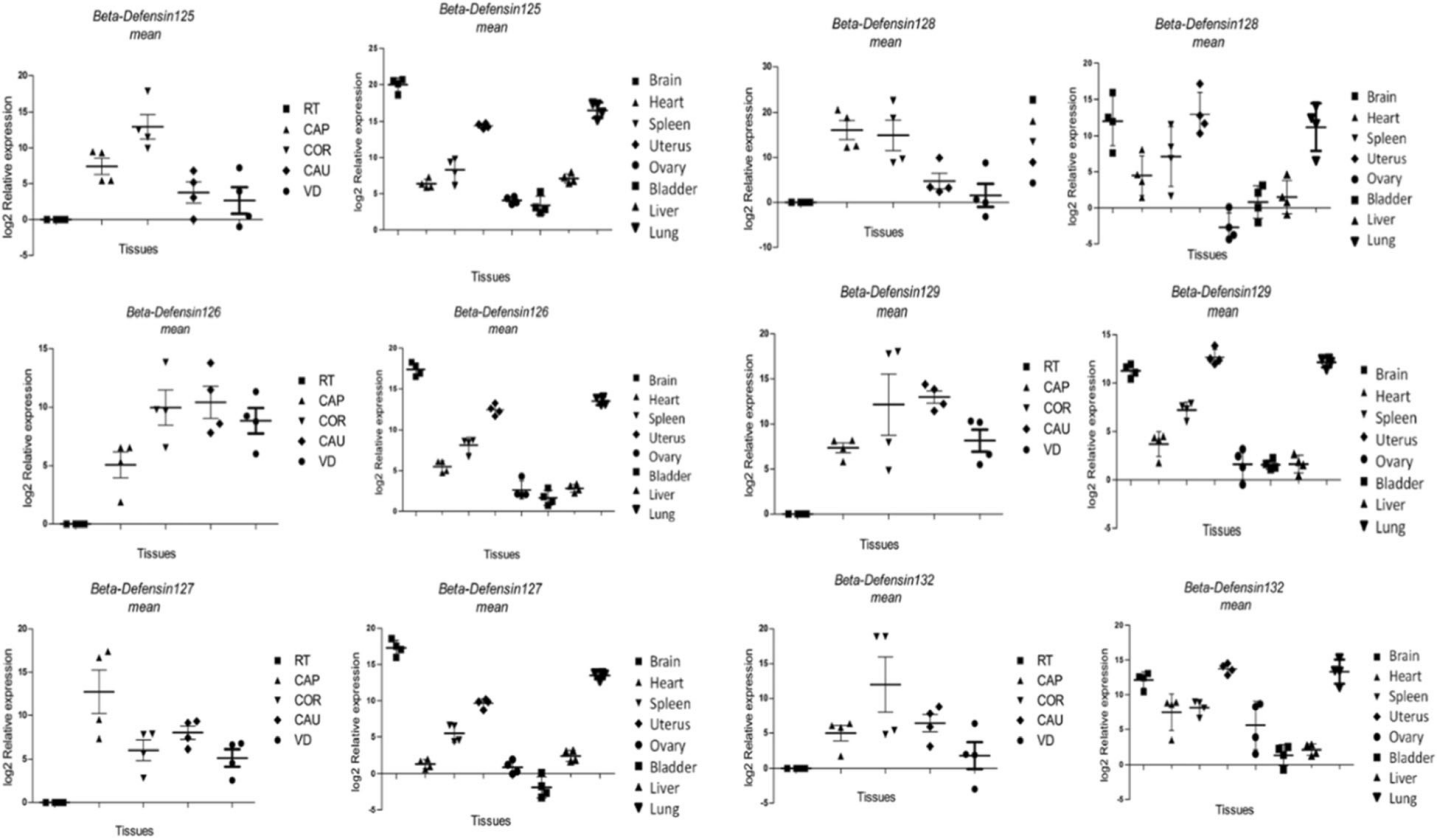
Pattern of expression of class-A BuBDs

The expression of the six CA-BDs was validated not only in the male reproductive tissues but also in non-reproductive tissues as well as female reproductive tissues of the buffalo. The variability among the biological replicates in the expression levels of BuBDs was comparatively lesser in the non-reproductive tissues than that of the male reproductive tissues. However, the disparate patterns of preferential spatial expression of BuBDs were present even in the non-reproductive tissues with the highest expression observed in the brain (Fig. 4, and Additional file 3). Particularly, the highest expression of BuBD-125, 126 and 127 (*P* < 0.001) was observed in the brain followed by the lung. Similarly, the expression of BuBD-128, 129 and 132 (*P* < 0.01) was also elevated in the brain followed by the uterus and the lung. The ovary and the bladder were the two tissues exhibiting the least levels of the expression, while an intermediate expression level was observed in the uterus for most of the class-A BuBDs. These observations surprisingly provided a clue that in contrast to the cattle, the buffalo female reproductive tissues actively expressed CA-BDs.

Relative expression profiles of the CA-BD genes across the various segments of the MRT and other body tissues in Murrah bulls & buffaloes. Expression values are normalized to GAPDH & eEf-2, represented by 0 on the graph. Horizontal bars represent mean and error bars represent the standard error of mean (SEM). Tissues are identified on the X-axis. RT = rete testis, CAP = Caput epididymis, COR = Corpus epididymis, CAU = Cauda epididymis, VD = Vas deferens. Y-axis represent relative expression levels.

## Discussion

The present study was undertaken to identify the cattle BD orthologs for characterization and evaluation of their sequence, gene ontology and physicochemical, structural characteristics of the translated products of class A BuBDs. In addition, the selection pressure characterization and the expression analysis of class-A BuBDs in the MRT and other body tissues were also performed to predict their reproductive functional significance. Thirty cattle BD orthologs, including the CA-BDs, were mined from the genome assemblies of the ruminants, including buffalo, pseudo-ruminants and non-ruminants. Despite the low sequence homology, the buffalo CA-BDs possess an analogous residue profile to their orthologs, thus have maintained the structural core which is required for their functions. The investigated CA-BDs were found to possess a similar molecular framework and secondary structural elements as reported in other BDs. Among CA-BDs, the BuBD-129 appears to be the novel functional ortholog of the primate or rodent BD-126. The diversity in the BD arsenal of buffalo immune system appears to be a result of contrasting selection pressures acting on their gene elements at different timescales.

The ensembles of microorganisms present in and on multicellular animals and their bodily fluids vary according to the physiochemical milieu in which they flourish [50, 51]. These pathogens drive the molecular evolution of their animal hosts by antagonistic co-evolution [52]. For example, the ruminants developed sophisticated immune mechanisms by their co-evolution with the rumen microflora [7]. Accordingly, a positive correlation exists between the, dynamic microbial insults in the niches inhabited by the host animals and the number BD genes found in a species [53]. We have mined 30 BD sequences (RNA & CDSs) in the buffalo, including 15 novel BDs, along with classical AMPs (LAP, TAP, EBD, etc.) that represents a large expansion of the buffalo BD repertoire comparable to that of the cattle. Our results indicated a rapid evolution of these genes in the buffalo through duplications usually followed by their diversification of their sequence and function, as reported in the cattle and other mammalian species [54]. This swift-evolution, considering their pleiotropic roles and the 3-D structural similarities despite the sequence dissimilarities, indicate diverse immune and non-immune functions of the buffalo CA-BDs. The class-A BuBDs considered in this study were predicted to possess antimicrobial activity by the SVM classifier. This indicates the putative conservation of the primordial antimicrobial function of the BDs in spite of attaining novel additional functions after episodic/pervasive diversifying selection [39, 55]. This prediction is underpinned by our finding that the CA-BDs were found to be expressed in a variety of non-reproductive tissues of the buffalo apart from the MRT.

All the class-A BuBDs were found to retain the canonical six cysteine motif, “X2–10CX5–6[G/A] XCX3–4CX9–13CX4–7CCXN”, in addition to a characteristic sequence feature the G-X-C motif which is required for their proper folding [56]. The class-A BuBDs contain ample basic residues such as lysine and arginine, which appears to help them in interacting with the negatively charged plasma membranes of microorganisms by electrostatic interactions [57]. The amino-acid compositional bias, in terms of the reduced share of the order-promoting residues and the prevalence of polar amino acids, substantiates the prediction of loops in the major region of these peptides which must render a substantial surface area of these BDs accessible to the surrounding solvent. The presence of the six-cysteines in a conserved molecular framework and the predicted presence of three β-strands in the anti-parallel arrangement suggest that the information for proper folding indeed exists in the primary sequence of the class-A BuBDs [58]. The three disulfide bonds stabilize the structural properties of the characteristic ‘β-fold’, which is vital to their molecular function/s. The increasing dissimilarity between the translated CA-BD gene products with the increasing evolutionary distance has had a comparatively lesser effect on the 3-D structure of buffalo CA-BDs. This explains why the ‘hallmark 3-D structure’ of the BDs was predicted to tolerate substitutions with residues of diverse physicochemical properties at most positions. However, not at the sites which are required for proper folding [59] e.g. the six cysteines and their neighbouring sites, as observed from the SNAP2 results for class-A BuBDs.

The focus of the recent studies has shifted from the canonical antimicrobial role played by these HDPs to their pleiotropic functions, for example, in the male reproductive processes where they assist the sperm to ultimately fertilize the oocyte. The major SSR events, which are necessary not only for the survival of the sperm in the FRT (e.g. immune-evasion) but also for the capacitation and fertilization with the oocyte, take place in the epididymis. The presence of the buffalo CA-BD transcripts in the MRT indicate their secretion in the epididymal lumen of the buffalo. Furthermore, the glycan modifying enzymes e.g. glycosidase and glycosyl-transferase are considered critical for the SSR events. Their family members transform the glycocalyx of the molecules involved in fertilization-specific activities which are also known to embellish the sperm-surface e.g. addition of sialic acid to DEFB-126 [38, 34], and [60]. The spermatozoa require DEFB-126 to penetrate the cervical mucus [42], to evade the immune response mounted in the FRT [43], and to form the oviductal sperm reservoir [14, 61]. Moreover, a small deletion (2 nucleotides) in the DEFB126 gene that modifies its O-glycan structure and thus the sperm surface was found to be implicated in sub-fertility in the human cohorts [62]. The terminal sialic acids present abundantly on DEFB-126 not only delay, but also assist the sperm in the capacitation process as and when required in the FRT [63]. Intriguingly, a novel CA-BD, BuBD-129 was predicted to be the most heavily glycosylated CA-BD family member rather the BD-126 as reported in primates and rodents [42]. It is thus legitimate to hypothesize that BuBD-129 could be the functional ortholog of the BD-126 in humans (Primates) and mice (Rodentia) primarily because of its preferential expression in the distal segments of MRT, a high potential of O- and N-glycosylation in its translated gene products, its gene length, chromosome cluster, extended tail as reported in the DEFB-126.

The BDs are primarily secreted by the epithelial cells of the gut, skin, airway, mouth, kidney, nose, eyes and mammary glands. However, the CA-BDs have been identified majorly in the epididymal luminal proteome that binds the sperm surface [45, 48, 64–67], and [68]. The buffalo CA-BDs were predicted to be secreted in the extracellular space, possibly into the epididymal lumen of the distal segments of the MRT. This was substantiated by the presence of the polar, hydrophilic amino acid residues, prediction of the loops as the major, secondary structural elements and the expression of their genes in the major non-reproductive tissues. Recently, most of the CA-BD genes (BBD125, 126, 127 and 128) have been characterized along with their genetic variants, which have now been implicated in the cattle bull sperm function [40].

In the present study, the comparison of CA-BD genes across the selected mammalian species revealed an evolutionary pattern, in which the different BD genes appeared to follow different evolutionary paths, albeit at different evolutionary time scales. Selection pressure characterization of the CA-BD genes revealed ample evidence of episodic diversifying selection in the ruminants, including the buffalo. Many immune-related genes have revealed evidence of directional or diversifying selection as they have to counter a variety of pathogenic fauna and gut flora which are evolving at high rates of genetic drift and shift [7, 69–71], and [72]. Afferent molecules, which are involved in sensing and recognizing the pathogens, are required to distinguish and thus help to eliminate a variety of pathogens. Such molecules have to adapt to the dynamic pathogenic fauna and microflora in their niches. Therefore, the afferent molecules are found to be under diversifying selection pressure. A relatively strong episodic positive selection pressure was found to have swept across the E2 and whole CDSs of the selected ruminants and the buffalo CA-BD genes. On the other hand, most ruminant CA-BDs were also found to contain at least one codon site under pervasive purifying selection. Efferent molecules which are involved in eliminating infections can’t afford to lose their specialized function, thus they are under purifying selection pressure [73]. The buffalo and ruminant CA-BDs considered in this study revealed evidence of episodic diversifying and pervasive purifying selection pressure on the E2 and the whole-CDSs which appears to be the driving force for sequence and functional divergence of the CA-BDs in the buffalo and possibly other ruminants. The CA-BD genes are now known to be implicated in male fertility in a number of species where they assist the spermatozoa to achieve their ultimate goal of fertilization [7, 30, 42, 46, 62, 63], and [74]. In fact, the evolutionary driver for innate immune genes could either be positive or negative selection, which depends upon their functional properties in the immune cascade [75]. The evidence of purifying selective pressure in the CA-BD gene elements also implies that the natural selection has had selected the most efficacious alleles because low tolerance for new variants is a common strategy for genes involved in the efferent arm of immunity [76]. Nevertheless, the evidence of diversifying selection pressure on these innate effector genes can’t be ignored because of its tendency to widen the spectrum of functional activity of these genes due to high rates of genetic drift, a signature of the innate immune system [77].

The observed patterns of selection of BD genes observed in this study, could have been moulded by the microbiome with which the given immune genes had co-evolved. Besides, factors like demographic features, and the microbial load of the host might also have important implications in deciding the direction and type of the selection [78]. Our results thus revealed that both the purifying [39, 55] and [78] and the diversifying selection [79, 80] have acted on the CA-BD genes, however, at different time scales. Although the major pervasive selection force in maintaining the crucial CA-BD alleles in ruminants appears to be the purifying selection, the evolutionary age of the BD genes should also be considered [81]. This is because of the duplication events, which appear to initiate a period of relaxed selection, where one of the duplicates maintains the wild type gene function and the other duplicate is free to explore a new functional space. Thus, if a new function is acquired then one of the duplicates will subsequently be maintained by purifying selection [82], as observed in the BuBD paralogs. It may also be possible that a BD gene earlier subject to positive selection may not be subject to such selective forces and vice versa due to the environmental dynamicity [79, 83]. The purifying selection force acting on the mature peptide (E2) and the whole CDSs of the ruminant CA-BD genes is expected to drive a low allele diversity as observed in some species [84, 85], while the diversifying selection force appears to have produced the diversity in the functional repertoire of these pleiotropic AMPs [79, 86]. Considering the contrasting selective forces, the CA-BDs appear to have evolved under an intricate balance of contrasting selective pressures and restraints, possibly a putative compromise between utilitarian selection (e.g. role in innate immunity) and sexual selection (e.g. role in reproductive processes).

The expression of class-A BuBDs, hitherto, has known to be restricted to the MRT and to be age-specific [7, 30], and [75]. However, constitutive expression of class-A BuBDs was observed in the buffalo non-reproductive and reproductive tissues in the absence of any visible infection. This indicates that BuBDs aren’t just evolutionary relics of their orthologs, but they are fully-fledged functional genes that are transcribed and probably translated too. Our observations differ from what has been reported in the cattle [7, 30], and [75] where their expression, including the BBD-126 was found to be restricted only to the MRT. Contrarily, some members of the CA-BD family, have also been reported in non-reproductive tissues of humans [65, 87], pig [88], rabbit [89] and even in the equine females [90]. The expression of class-A BuBDs in non-reproductive tissues apart from MRT tissues points to their active antimicrobial roles, which means that these defensins are still maintaining the primordial antimicrobial activity even though having gained new additional functions by diversifying selection. Further functional assays are required to validate their molecular functions. The expression of these genes in MRT had been reported to be absent in the MRT of immature cow bulls [30]. Interestingly, the protein product of the BBD-126 was later shown to be present in the testes of immature cattle bulls despite the absence of any transcription [91]. Further investigation is required to ascertain whether the protein BBD-126 is translocated from other tissues to the testes of immature cow bulls. As discussed earlier, until recently, the expression of the CA-BDs has been ascribed to specific sex, age and tissue. An interesting question that arises is “why is this specificity not observed in the buffalo?” Is it because of the differential composition of the ensemble of flora in the gut of the buffalo? Though the answer remains enigmatic, a clear picture has emerged, that the buffalo is as different from the cattle as are other ruminants with reference to the CA-BD genes. Our observations on the expression analysis of CA-BDs in the MRT indicated that the observed spatial variation in their expression point towards the variable biological functions at different sites [69, 91], and [92]. The spatial variation observed in the expression of these CA-BDs could be explained by the fact that the different BDs target only the region-specific pathogens in accordance with their anatomical distributions [56, 82]. The distal segments of the MRT (the cauda epididymis and the V.D.) are the regions that usually exhibit the highest expression levels of the CA-BDs [34, 35]. A similar trend was observed for the buffalo CA-BDs, especially for a novel candidate BuBD-129 in addition to the BuBD-126, which also had abundant transcripts in the distal segments of the MRT. This unusual expression pattern of the class-A BDs in the buffalo organs such as the heart, brain, spleen, and the epididymis indicated that BuBDs appeared to have evolved in coherence with the hypothesis of ‘Niche adaptation’ [7]. It is also likely that the observed high basal-level expression represents a response, which is induced in the context of colonization (tolerance to self and co-inhabitants), or any invisible infection thus being an immune advantage [50]. Infections or pathogenic insults are an important force driving the evolution of defensins and other immune genes. The high and the variable expression of the class-A BDs in the buffalo MRT without any visible infection could also be indicative of their putative role in assisting the sperm to achieve their goal of fertilization, which has also been established to be implicated in bull fertility of cattle [40].

## Conclusion

The buffalo possesses a diverse catalogue of BD genes, which have maintained a direct orthologous relationship with several ruminant and non-ruminant species. The increasing dissimilarity between sequences with increasing evolutionary distance has had a comparatively lesser effect on the protein structure of class-A BuBDs. A complex interplay of contrasting selective forces acting on different evolutionary timescales has raised a diverse repertoire of the BD arsenal in the buffalo immune system. The expression pattern of class-A BuBDs observed in this study suggested that these molecules maintain an infection-free environment not only in the reproductive but also in the non-reproductive parts of the buffalo. The quantitative variation in the expression patterns in various tissues signifies that the site-specific expression pattern may be attributed to the differential functions performed by the class-A BuBDs in accordance with their anatomical distributions. A study is warranted to assay the antimicrobial and other pleiotropic activities of these defensins in the MRT or on the sperm-surface*.* As the annotation of the buffalo genome is presently in the primitive stage, a fully sequenced and annotated buffalo genome may lead to the thorough characterization of the BuBDs, the discovery of additional orthologs and elucidation of their functional attributes.

## Methods

### Experimental design

The RNA sequences and complete CDSs of *Bos taurus* BDs (retrieved from assembly ARS-UCD 1.2) were used to mine their sequence orthologs in the latest, chromosome level-RefSeq assemblies of buffalo and 15 other species including true-ruminants, pseudo-ruminants and non-ruminants. The retrieved CDSs of the six CA-BDs were translated and subject to various in silico tools to evaluate their physicochemical properties and some post-translational modifications and to perform the sequence ontologies of the CA-BDs. The CA-BDs, known to play crucial roles in reproduction, were further used for evolutionary (selection pressure characterization), structural and expression pattern analyses in *n* = 4 biological replicates in five segments of the MRT (Rete testis, caput, corpus & cauda epididymis, and vas deferens) and eight other body tissues (Brain, heart, spleen, bladder, uterus, ovary, lung and liver).

### In silico sequence retrieval & analyses

Fifty-seven beta-defensins (BDs), maximum in any ruminant species, have been reported in *Bos taurus* [48]*,* therefore it was considered the reference-species for searching orthologs in buffalo and other selected mammalian species. The RNA/CDS sequences for *Bos taurus* BDs were mined from its latest RefSeq assembly (ARS-UCD 1.2). These RNA/CDS were subjected to BLASTN [93] against the latest RefSeq assembly of *Bubalus bubalis* (UOA_WB_1) and other ruminants and non-ruminant species. The sequences were obtained from GenBank [94], if not found in respective assemblies. The assemblies of ‘chromosome level (if available) were used to determine the loci, genome coordinates, duplications, and orientation of the genes and gene elements.

The retrieved CDSs were translated using the ExPASY translate tool [95]. The sequence characteristics & ontology, secondary structure, composition, solvent accessibility etc. of the class-A BuBD proteins (Chromosome 13 defensin cluster) were predicted by the various tools as implemented on the Predict protein server [96]. The functional effects of various mutations/SNPs were predicted by SNAP2, a trained classifier that not only takes the sequence and variant features into account, but also the evolutionary information from an automatically generated MSA [97]. The protein disorder in the class-A BuBD proteins was predicted by Meta-disorder predictor method [98]. It is a combination of several orthogonal methods that captures many types of disorder, combining the output from various prediction methods with sequence profiles and other useful features such as predicted solvent accessibility, secondary structure and low complexity regions. The Gene Ontology analysis for the BuBDs was performed by Metastudent, which predicts the gene ontology (GO) terms for protein sequences through homology [99]. The presence of the Protein-Protein Interaction, PPI and Protein-Nucleotide Interaction, PNI was predicted through the interaction sites identified by profISIS [100] and someNA [101] tools. The neural network predictions of mucin type GalNAc O-glycosylation sites were produced using NetOGlyc 4.0 server [102] whereas N-glycosylation sites were predicted using artificial neural networks that examine the sequence context of Asn-Xaa-Ser/Thr *sequons* using NetNGlyc 1.0 server [103]. The predictions for the presence of a transmembrane helix were made using TMHMM server v.2.0 [104]. Since the BDs are known to be HDPs, an in silico prediction of the presence of the antimicrobial activity was done using Support vector machine classifier algorithm available at the CAMP_R3_ database [105]. The presence of the signature G-X-C motif was inspected manually in the CA-BDs.

### Structure analysis

Very few structures of BDs exist in Protein Data Bank-PDB [106], although a search for the term ‘beta-defensin’ yields 74 results. However, only ten of them are mammalian defensins and the only ruminant BD listed is BNBD-1, the bovine neutrophil beta-defensin. Thus the class-A BuBDs could be modelled only on the existing BD structures in the PDB. The BuBD-125,126 and 132 were modelled on human BDs namely hBD6 (2lwl.1.a), BuBD-127 on hBD4 (5ki9.1.A) respectively, whereas the BuBD-128 was modelled on hBD1 (1kj5.1.a) and BuBD-129 on the bovine neutrophil defensin, BNBD (1bnb.1.a). The structural features of the CA-BDs were analysed using the folding patterns of these peptides as predicted by the SWISS-MODEL homology-modelling sever [107]. The PDB files of the models were viewed with UCSF-Chimera *ver.* 1.13.1 [108]. The structural assessment of the models was done using Molprobity *ver.* 4.4 [109].

### Evolutionary analyses: Orthologs and Paralogs

The retrieved class-A BuBDs were compared with their orthologs in other ruminants (*Bos taurus, Bos indicus, Brahman cattle, Bos mutus, Bison bison, Ovies aries, Capra hircus, Odocoileus texanus*, pseudo-ruminants (*Camelus dromedaries & bactrianus*), monogastric organisms (*Felis catus, Canis familiaris, Loxodonta africana, Oryctolagus cuniculus*) and primates (*Homo sapiens*). The respective CDSs were aligned using multiple sequence alignment (MSA) on MAFFT *ver.* 7.409 [110] and were visualized using Jalview 2.10.5 [111]. The MSAs obtained from MAFFT program were visually inspected and subsequently subjected to GBlocks filter on translator server [112] to check for ambiguous sequence regions. The cleaned alignments were used for further analysis.

### Phylogenetic tree

The whole-CDSs of class-A BuBDs along with their exons viz. the E1 and the E2 were aligned using clustal in Mega X [113]. As a preliminary step for phylogenetic studies, the Maximum likelihood method (MLM) fit for 56 different amino-acid substitution models and 24 different nucleotide substitution models [113, 114] were obtained. The best model describing the substitution pattern was selected and was used for subsequent analyses. The evolutionary analysis for phylogenetic tree building was done by the MLM method in conjunction with the best-chosen model. The bootstrap consensus tree inferred from 1000 replicates was taken to represent the evolutionary history of the analyzed taxa [115].

### Selection pressure characterization

As a pre-processing step for inferring selection pressure, all CDSs for CA-BD genes were checked for evidence of intragenic recombination using GARD [116]. The significant topological incongruence of recombination breakpoints was tested using the Kishino–Hasegawa test [117]. For characterizing the patterns of selection on these sequences, statistical models and tools from the HyPhy (Hypothesis testing using Phylogenies) package implemented in the accompanying datamonkey web-server [118] were used and *P*-values/posterior probabilities of 0.05 were considered significant. The selection pattern characterization was also performed using molecular evolutionary genetic analysis tool MEGA-X.

#### Strategy 1: analysis of selection pressure on the CA-BD orthologs by HyPhy

The cleaned alignments for E1, E2 and whole-CDSs of class-A beta-defensin genes were used for the analysis on HyPhy. The testing of branches of interest viz. *Bubalus bubalis* or ruminants, for the episodic positive/purifying selection, was performed using aBSREL [119]. aBSREL is a better version of the common branch-site models and is a preferred approach for identifying positive selection at individual branches since it models both, site-level as well as branch-level ω (dN/dS) heterogeneity. The shifts in the stringency of natural selection in the CA-BD genes were determined using a hypothesis testing framework RELAX [120]. The branch with buffalo or all true ruminants as taxa was a priori*’* selected as the foreground *vis-à-vis* the background branches branch to be tested for section pressure characterization. Determination of pervasive (at a subset of branches) positive or purifying selection was done by FEL [121] which uses a maximum-likelihood (ML) approach to infer non-synonymous (dN) and synonymous (dS) substitution rates on a per-site basis. However, for determining episodic positive or purifying selection in the CA-BD sites, MEME was used [122]. For each site, MEME infers two ω rate classes and corresponding weights representing the probability that the site evolves under each respective ω rate class at a given branch. Lastly, the gene-wide test for positive selection in the CA-BDs by asking “whether a CA-BD gene has experienced positive selection at any site on at least one of the branches” was performed by BUSTED (Branch-Site Unrestricted Statistical Test for Episodic Diversification) [123]. The parent branch with the buffalo or the ruminants as taxa were ‘a priori*’* selected as the foreground branch to be tested. For each phylogenetic partition (i.e. the foreground and background branch sites), BUSTED fits a codon model with three rate classes, constrained as ω1 ≤ ω2 ≤ 1 ≤ ω3. BUSTED simultaneously estimates the proportion of sites per partition belonging to each ω class.

#### Strategy 2: analysis of the selection pressure on the CA-BD orthologs by molecular evolutionary genetic analyses (MEGA-X)

Additionally, to test whether the sequences have evolved with a similar pattern of substitution, a Monte Carlo test (1000 replicates) was used to estimate the *p*-values for the disparity index test, indicating the differences in base composition biases between sequences [113, 124]. The codon-based Z-test of neutrality (dN = dS), positive selection (dN > dS) and negative selection (dN < dS) for analysis between sequences as well as averaging over all sequence pairs were conducted for assessment of selection pressures [113, 125]. The test statistic used for detecting the codons that have undergone positive selection was dN – dS, where dS is the number of synonymous substitutions per site (s/S) and dN is the number of non-synonymous substitutions per site (n/N). Moreover, the one-tailed Fisher’s exact test for neutrality was also conducted for both the exons E1 and E2 as well as for complete CDS sequences [113, 125], and [126]. To further corroborate the results the population genetic test statistic Tajima’s D was also calculated [113, 127].

### Analysis of selection pressure on the CA-BD paralogs by HyPhy

To characterize the driving force behind the functional diversification and maintenance of the buffalo BD genes, we tested the role of Darwinian selection. Thus, the retrieved CDSs of the paralogs from buffalo were subjected to the same tests used for the orthologs using the HyPhy package.

### Expression dynamics of the buffalo CA-BD genes across the MRT and other tissues

#### Tissue collection

The MRT and other body tissues were collected in the month of January of 2017 from a local abattoir, Delhi (28°37′40″N, 77°19′51″E) with the average temperature varying from 7^o^-10 °C. The five segments from the MRT viz. the caput, corpus and cauda epididymides, the vas deferens and the rete testes and eight body tissues viz. the brain, heart, spleen, uterus, ovary, bladder, liver and the lung were collected aseptically within 20 min of slaughter to obtain 3 mm sized samples from healthy and mature Murrah buffalo bulls (*n* = 4 biological replicates) aged between 3 and 4 years. The tissue samples were immediately stored in the RNA later solution (Ambion, USA) and afterwards stored in -80 °C. The maturity and age of bulls were confirmed by the presence of spermatozoa in rete testes giving it a milky white appearance and counting of teeth prior to slaughter respectively.

### RNA isolation

Total RNA was isolated from the MRT tissues using TRI Reagent, RNA isolation reagent (Sigma-Aldrich, USA) and quantified using a NanoDrop ND-1000 UV–Vis spectrophotometer (NanoDrop Technologies Inc., Wilmington, DE, USA). The A_260/280_ and A_260/230_ were close to 2.0 for all the samples used in the study. The quality of the extracted RNA was assessed by running 200 ng of the RNA (heated at 70 °C for 1 min) in non-denaturing TAE buffered 1.2% agarose (Sigma-Aldrich, USA). The isolated RNA was given the DNase I (Thermo Scientific, USA) treatment, according to the manufacturer’s instructions to get rid of gDNA contamination.

### cDNA synthesis

RevertAid, H Minus first-strand cDNA synthesis kit (Thermo Scientific, USA) was used to convert 2 μg of RNA into cDNA as per manufacturer’s instructions. Briefly, 2 μg of RNA was mixed with oligo-(dT)_n_ and random hexamer primers and subsequently the nuclease-free water was added. The reaction mixture was incubated at 65 °C for 5 min. Later, the reaction buffer, RNase inhibitor, dNTP mix and the RevertAid H minus M-MuLV reverse transcriptase were added to make final reaction volume of 20 μl and incubated at 25 °C for 5 min, followed by incubation at 42 °C for 1 h in a thermal cycler (Biometra, Analytik Jena, Germany). The reaction was terminated by heating the mixture at 70 °C for 5 min. A reverse transcriptase negative control was prepared alongside for the assessment of residual genomic DNA contamination in the RNA sample.

### Primer design

The primer designing tool at NCBI, the Primer-BLAST [128] was used to design primers for buffalo CA-BD genes viz. BuBD-125, 126, 127, 128, 129 and 132 and three reference genes viz. HPRT (Hypoxanthine-guanine phosphoribosyltransferase), eEF-2 (eukaryotic elongation factor 2) and GAPDH (glyceraldehyde-3-phosphate dehydrogenase) from conserved regions found in MSA (MAFFT *ver.*7.409) [110]. Intron-spanning primers, wherever possible, were designed. However, the HPRT was later excluded from the investigation as the C_q_s obtained were greater than 38 in three out of five MRT tissue samples. The self-annealing sites, and the mismatches and the secondary structures in the primers were checked using Oligo Calc [129]. The specificity of primers was again checked using the BLAST alignment tool [95] and in silico PCR [130, 131] was run for each set of primers prior to commercial synthesis (Sigma-Aldrich, USA).

### RT-qPCR optimization

To ensure the quality of RT-qPCR and RT-qPCR data MIQE guidelines [132] were followed at every step, wherever possible (Additional file 4).

### RT-qPCR assay

The relative quantification of the BD genes was done on a Roche LC-96 light cycle platform using the Maxima SYBR Green qPCR master mix (Fermentas, USA) in a 10 μL reaction mix. The thermal profile was 95 °C for 10 min, 40 cycles consisting of denaturation at 95 °C for 15 s, annealing at variable optimized temperatures for 20s and extension at 72 °C for 20 s, followed by the melt curve protocol with 10 s at 95 °C and then 60 s each at 0.5 °C increments between 65 °C and 95 °C. A no-template control (NTC) was run in each plate to confirm the absence of nucleic acid contamination. A melt curve analysis was performed to ensure a specific, unique product formation and to ascertain minimal primer-dimer formation. The RT-qPCR products were subjected to agarose gel electrophoresis for ensuring specific product formation.

### RT-qPCR data and statistical analysis

The mean sample C_q_ (Cycle of quantification) values for different transcripts were calculated for duplicate samples and relative expression ratio of the beta-defensin gene expression was calculated using the ΔΔC_t_ (Cycle threshold) method [133], compared with the average of the two reference genes, glyceraldehyde 3-phosphate dehydrogenase (GAPDH) and Eukaryotic elongation factor 2 (Eef2). Here, ΔC_t_ is the difference in Ct between target and reference and ΔΔC_t_ is the difference in ΔC_t_ between all the samples and the calibrator, the sample with the lowest expression. The Rete testes were taken as the calibrator (control) tissue since the defensin genes are not known to be expressed in the rete-testes. They are known to start expressing from the caput epididymis continuing through vas deferens. The differential gene expression levels among the tissues were examined for normality of distribution, transformed where appropriate using log transformation, and analysed by ANOVA and Tukey post-hoc test as implemented in GraphPad Prism 5.0 (for Windows, GraphPad Software, La Jolla California USA, www.graphpad.com) and a *P*-value <0.05 was considered to be statistically significant. The Kruskal-Wallis non-parametric one-way ANOVA was used to measure the heterogeneity quantified transcripts in bulls & cows. For visualization of clustering of data based on relative expression, a heat map was plotted by the Clustvis rendering tool [134] using the means for all the replicates for all tissues & genes, to depict the expression pattern of BuBDs across MRT and other body tissues.

## Supplementary information

**Additional file 1.** Orthologs of BDs. CDS & protein sequence data for 30 BDs in all the species. The data for gene loci, coordinates, orientation etc. is available on request.

**Additional file 2.** Assemblies & novel defensins. CDS & protein sequence data for those BuBDs whose information i.e. the mRNA, CDSs aren’t yet listed in the Genbank alongwith the latest RefSeq assemblies from where the sequences were mined.

**Additional file 3.** Results from the MSA (Figure S1) & various tools implemented on the predict-protein server (Figure S2-S7). Also a pictorial representation of the mean expression profiles of these BDs in buffalo tissues (Figure S8).

**Additional file 4.** qRT-PCR optimization. The protocol followed for standardization of the qRT-PCR for the six class-A BuBDs in accordance with MIQE guidelines.

## Data Availability

All data generated or analysed during this study are included in this published article and its supplementary information files.

## Abbreviations

AMPs: Antimicrobial peptides
E1: Exon 1
E2: Exon 2
BD/ DEFB: Beta-defensin
BuBD: Buffalo beta-defensin
BUSTED: Branch-Site Unrestricted Statistical Test for Episodic Diversification
CA-BD: Class-A beta-defensin
CCR: Chemokine receptor
CR: Conception rate
EBD: Enteric beta-defensin
eEF2: Eukaryotic elongation factor
FEL: Fixed Effects Likelihood
FRT: Female reproductive tract
GAPDH: Glyceraldehyde-3-phosphate dehydrogenase
GARD: Genetic Algorithm for Recombination Detection
aBSREL: Adaptive Branch-Site Random Effects Likelihood
MEME: Mixed Effects Model of Evolution
GSM: Gene-specific motif
GSM: Gene-specific motif
CMP: Cervical mucus penetration
HDPs: Host defence peptides
HyPhy: Hypothesis testing using Phylogenies
LAP: Lingual antimicrobial peptide
MEGA: Molecular Evolutionary Genetic Analyses
MLM: Maximum likelihood method
MRT: Male reproductive tract
MSA: Multiple sequence alignment
RT: Rete testis
SNP: Single nucleotide polymorphism
SPP: Seminal plasma protein
SSR: Sperm surface remodelling
SVM: Support vector machine
TAP: Tracheal antimicrobial peptide
VD: Vas deferens
