# Supplementary material for: Unusual interplay of contrasting selective pressures on β-defensin genes implicated in male fertility of the Buffalo (Bubalus bubalis)
Source: BMC Evol Biol. 2019 Nov 26;19:214. doi: 10.1186/s12862-019-1535-8 (PMC6878701; doi:10.1186/s12862-019-1535-8)
Supplement: Supplementary file 3 — Additional file 3. Results from the MSA (Figure S1) & various tools implemented on the predict-protein server (Figure S2-S7). Also a pictorial representation of the mean expression profiles of these BDs in buffalo tissues (Figure S8). [file 12862_2019_1535_MOESM3_ESM.docx]

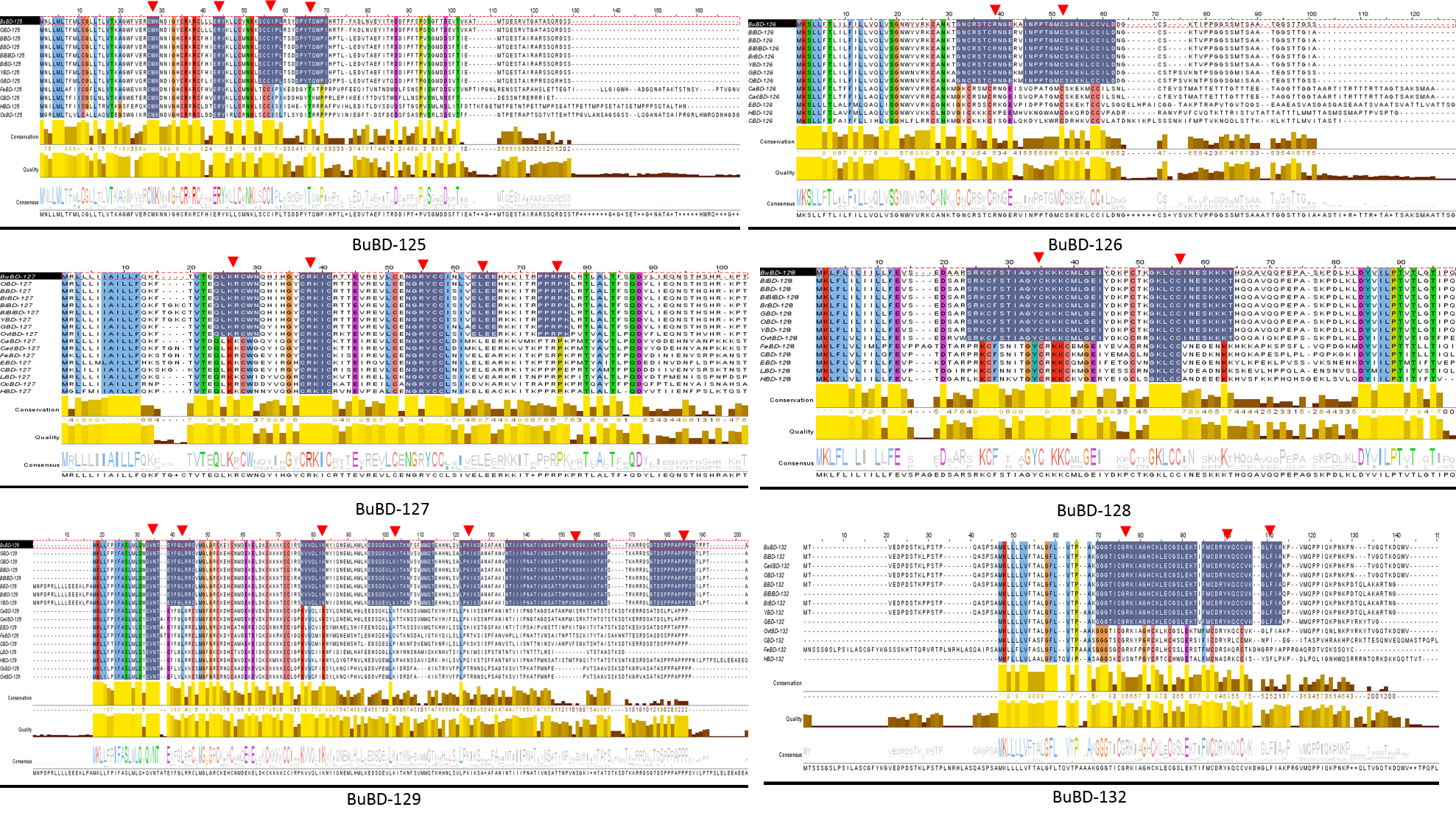


**Fig.** 1: Multiple Sequence Alignment of the retrieved CA-BD protein sequences of the buffalo, computed on MAFFT *v.*7.409, viewed using Jalview *v.*2.10.5 in Clustal colour scheme. Only the completely conserved sites are being colored. The beige colored boxes (red arrows atop) represent the defensin gene specific motifs (GSMs, see text)


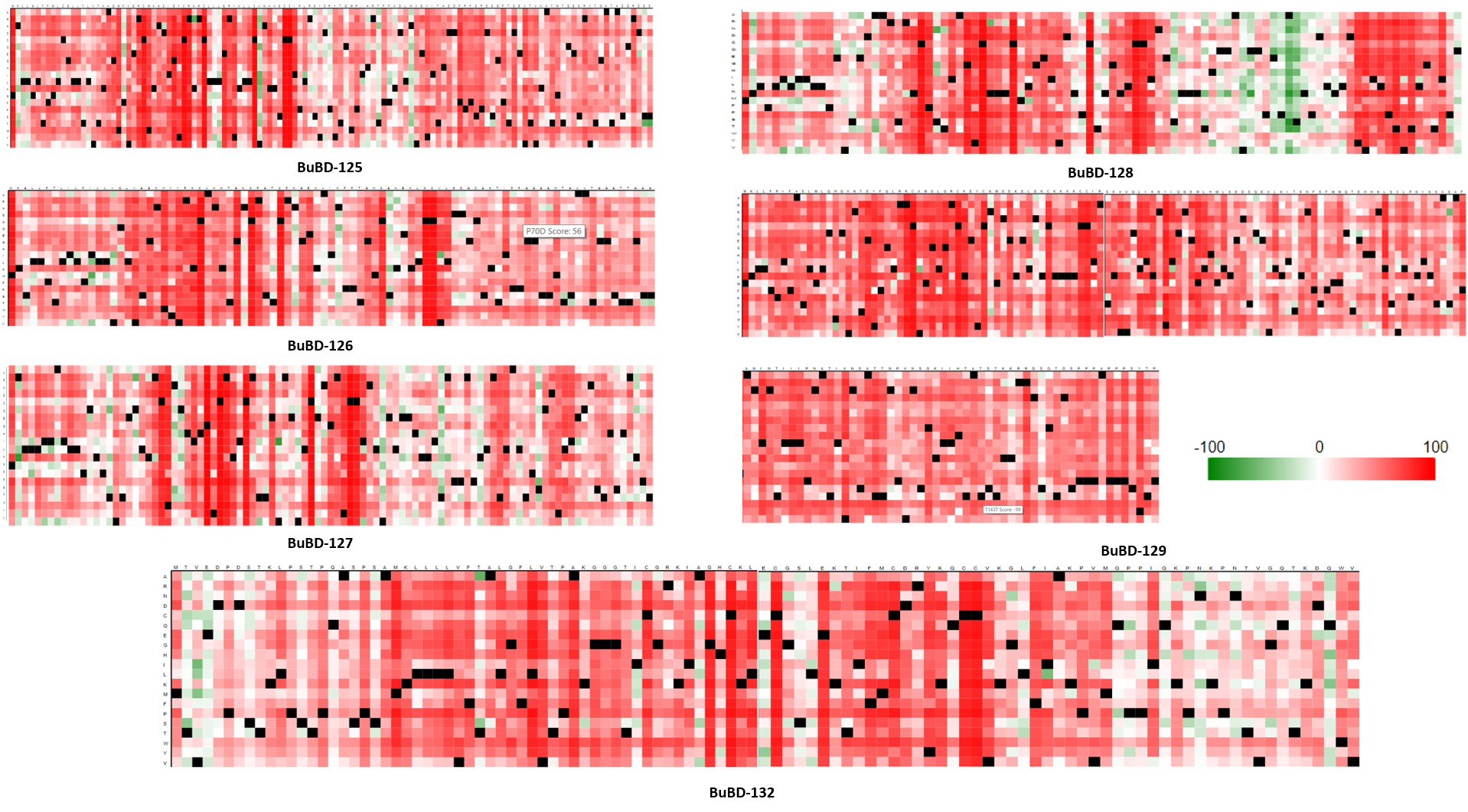
 **Fig.** 2: The heat map represents the functional effects of mutations, of the six class-A β-defensins of Buffalo, showing each substitution independently for each position of a protein. Dark red indicates a high score (score > 50, strong signal for effect), white indicates weak signals (-50 < score < 50), and green a low score (score < -50, strong signal for neutral/no effect). Black marks the corresponding wildtype residues


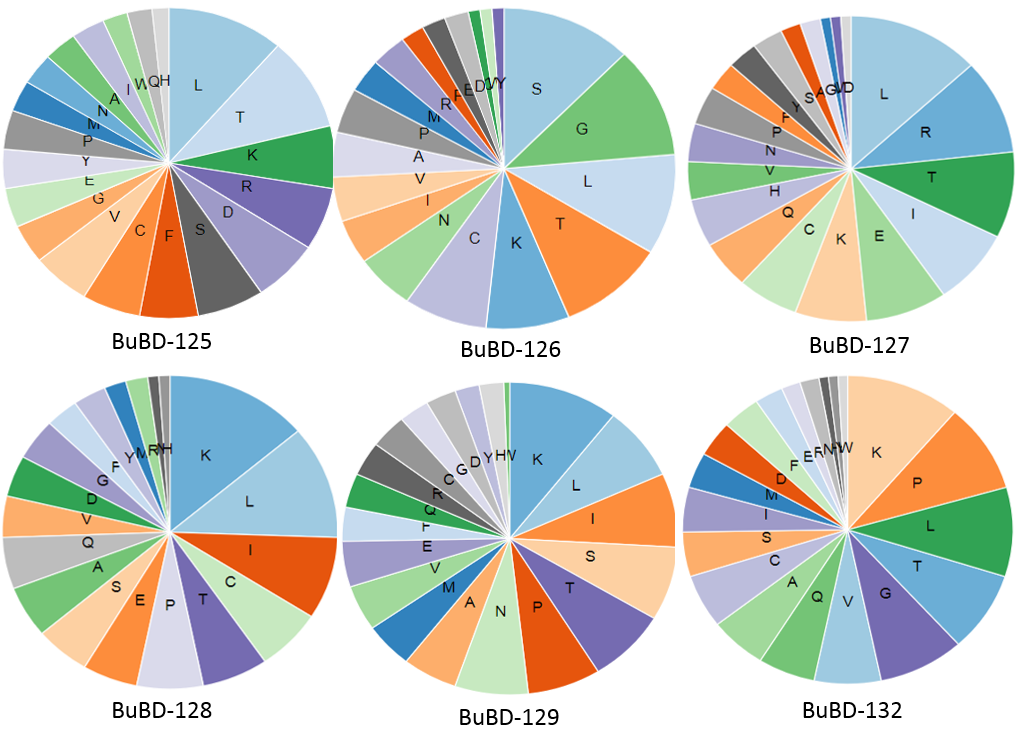


**Fig.** 3: The amino acid compositions of the six CA-BDs of the Buffalo


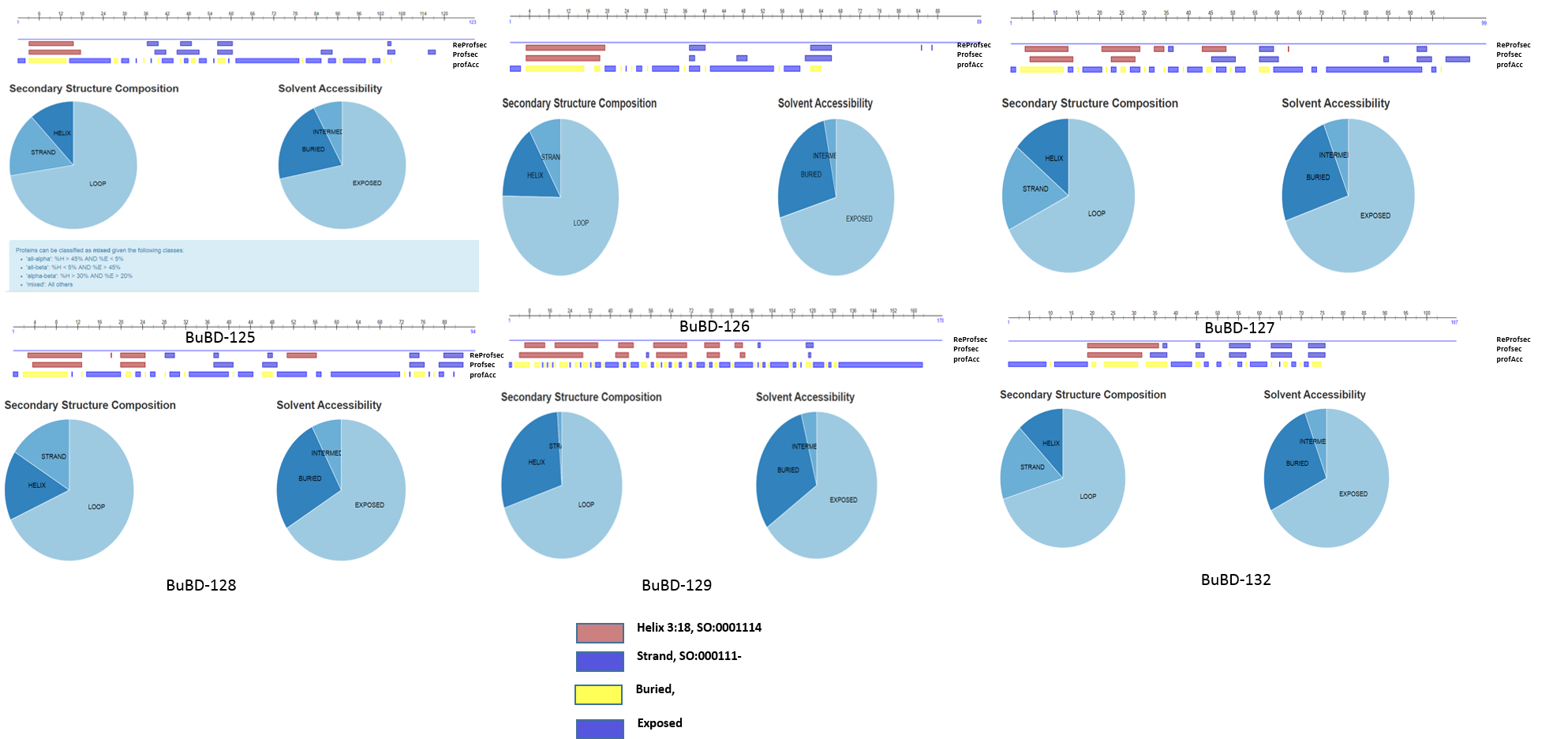


**Fig.** 4: Secondary structural element and solvent accessibility profile of the six class-A β-defensins of Buffalo as predicted by a system of neural networks using evolutionary information from Multiple Sequence Alignments, implemented in PROFsec & PROFacc on the Predict protein server


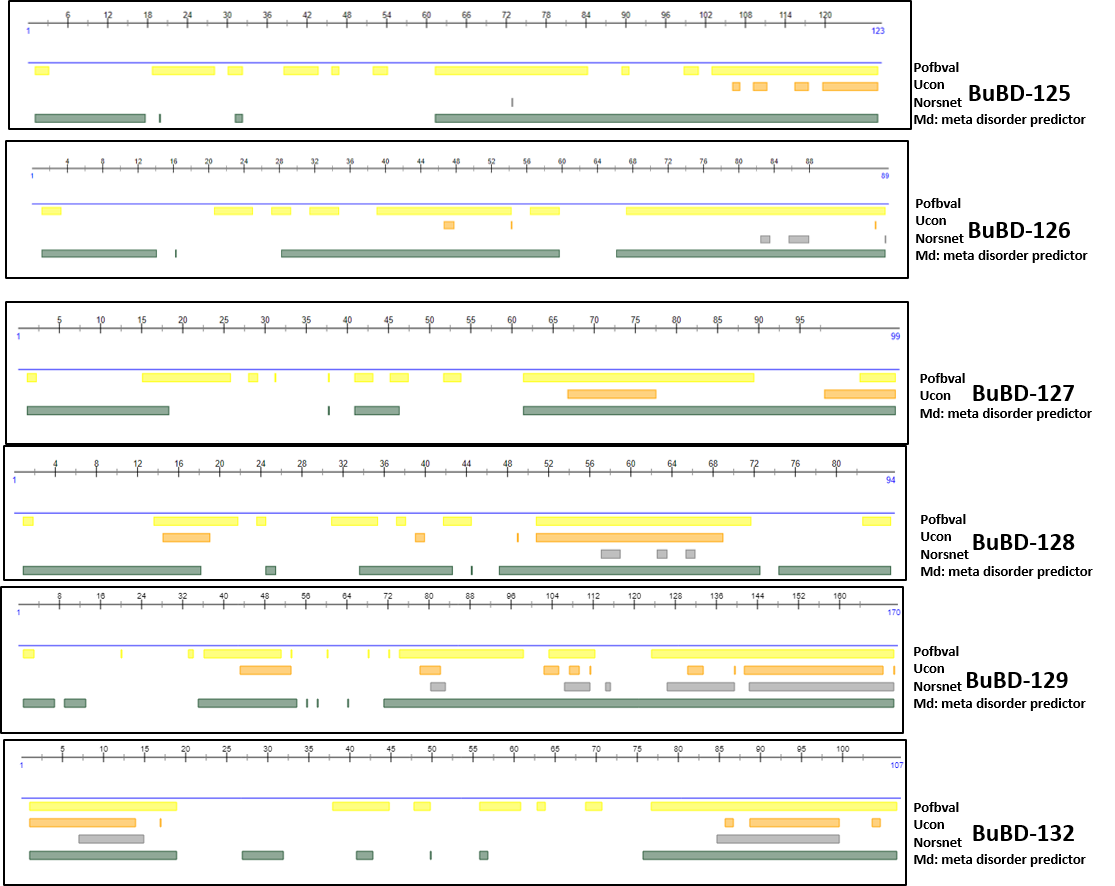


**Fig.** 5: The intrinsically disordered regions (IDRs) from the sequences of six CA-BD proteins predicted by Meta-Disorder (MD) using evolutionary profiles and sequence features correlating with disorder, depicting the output from several prediction methods


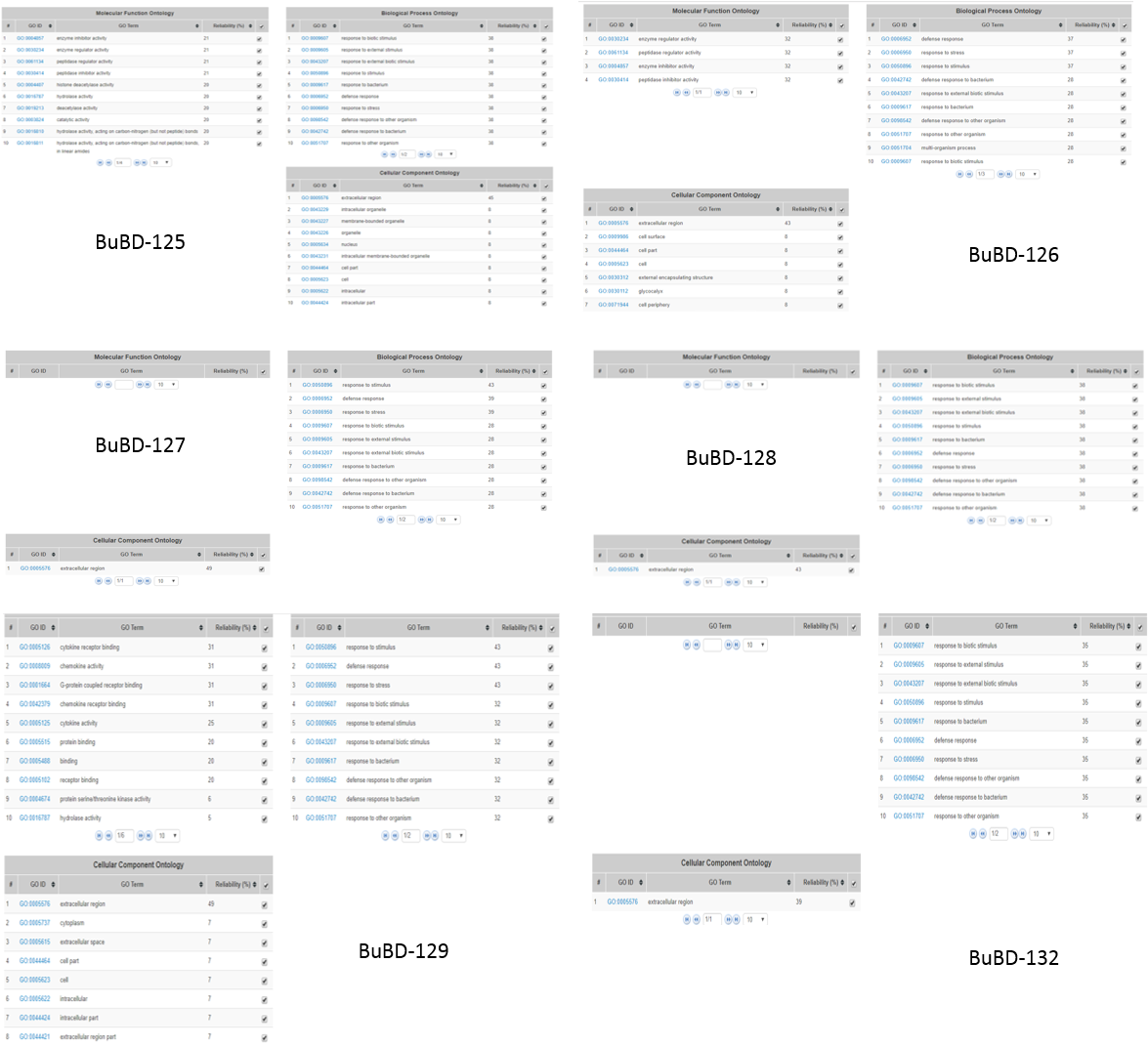


**Fig.** 6: GO terms’ predictions for the six CA-BD protein sequences of buffalo through homology based inference sets by Metastudent using the smart meta-classifier for calculating GO terms


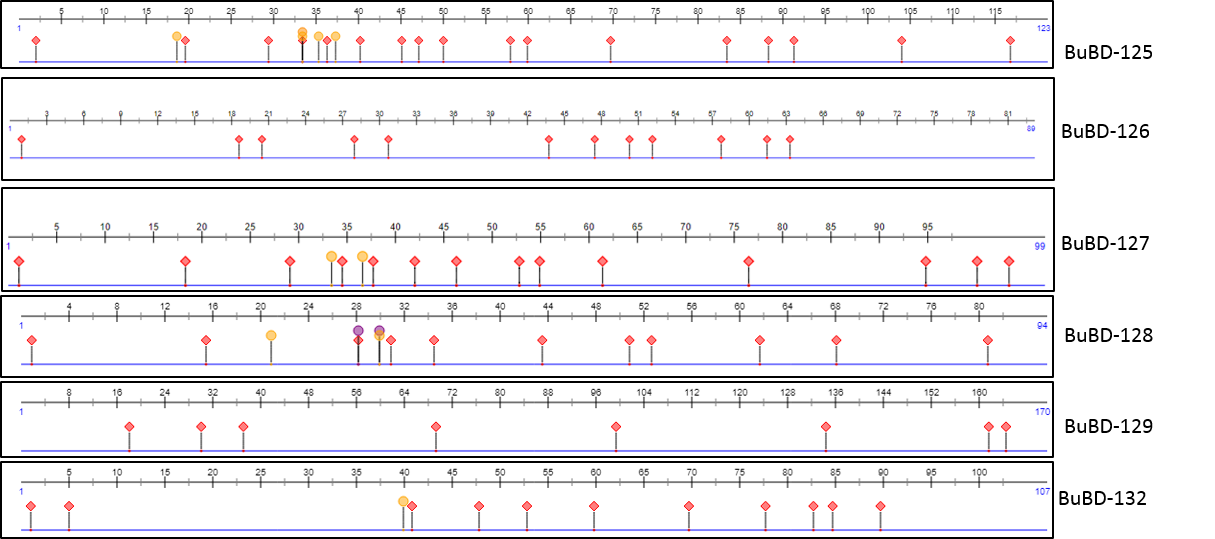


**Fig.** 7: The predicted Protein-Protein and Protein-NT interaction sites identified from the six class-A β-defensin protein sequences of Buffalo using predicted structural features (implicit 3D structural information) with evolutionary information and ANNs. Red diamonds indicate protein binding region: SO-0000410 while yellow circle indicates nucleotide binding region: SO 0001429


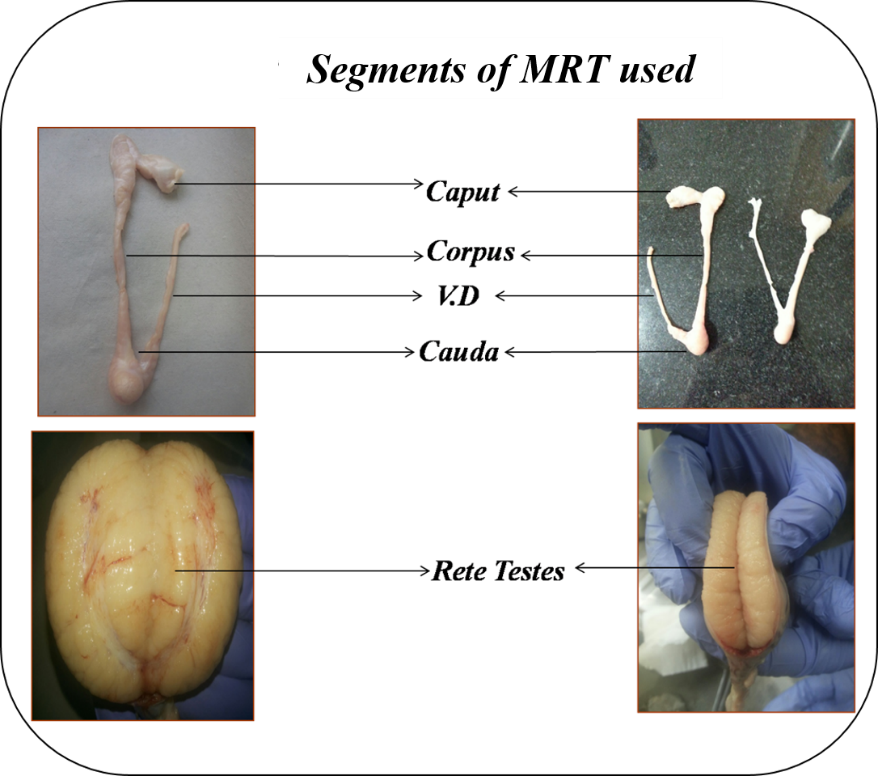


**Fig.** 8 Representative images of the five sample tissue sections of the MRT (n=4) used in this study viz. Rete testis, caput, corpus & cauda epididymis and vas deferens


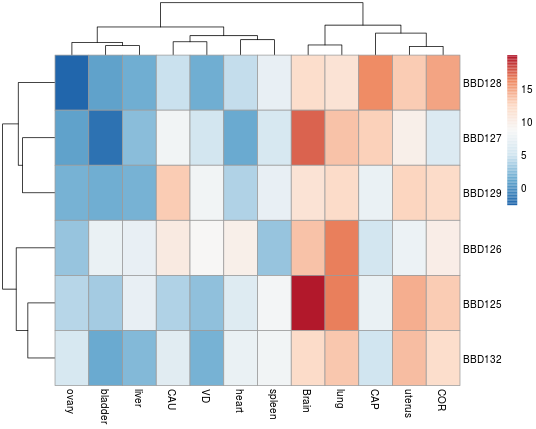


**Fig.** 9. Heat-map based on the mean relative expression profiles (n=4) of all the CA-BD genes in all the tissues considered in this study. No scaling was applied to rows. Rows and columns were clustered using Euclidean distance and complete linkage. Rows represent CA-BD genes, Columns represent tissues used in this study. Right vertical bar represents color key for mean relative expression dynamics across all the tissues
